# Adrenal Cortical Rests in the Fallopian Tube: A Case Report and Review of the Literature

**DOI:** 10.7759/cureus.27649

**Published:** 2022-08-03

**Authors:** Bayan Hafiz, Fatimah Alturkistani

**Affiliations:** 1 Department of Anatomical Pathology, King Abdulaziz Medical City (KAMC), Jeddah, SAU

**Keywords:** adenomyosis, ectopic tissue, hysterectomy, fallopian tube, adrenal rest

## Abstract

Ectopic adrenal rest is a rare phenomenon usually discovered incidentally during microscopic evaluation. The most common site reported in the literature is the genitourinary system and pelvis. Ectopic adrenal rest is more common in male than in female children. The documented site for females is mainly along the broad ligament. However, only two cases of ectopic adrenal rest in the fallopian tube have been reported in the literature, showing that they are extremely rare.

In this article, we outline a case of adrenal cortical rest that was discovered incidentally during a microscopic examination of the left fallopian tube after a total hysterectomy with a left salpingo-oophorectomy specimen from a 49-year-old female patient who was complaining of severe bleeding related to severe adenomyosis.

## Introduction

Ectopic adrenal tissue (EAT) is one of the rare entities discovered by Morgagni in 1740. The usual anatomical location of the adrenal glands is above the kidneys [1]. The presence of ectopias of the adrenal gland was reported in male children, appearing most commonly in the genitourinary tract, including the testis and pelvis [2]. In the English literature, only two cases were reported with the presence of ectopic adrenal cortical rest in fallopian tubes, indicating that it is extremely rare [3,4].

In this article, we report a case of an ectopic adrenal rest in a 49-year-old female patient, which was discovered incidentally during microscopic examination of a subtotal hysterectomy specimen that was excised due to extensive adenomyosis.

## Case presentation

The case involves a 49-year-old female nulliparous (Parity 0 + Gravida 12) with hypertension and recurrent pulmonary embolism. She presented to the emergency department complaining of menorrhagia. She had a regular cycle, but she noticed that the menses had been heavier than usual for the previous five months. The patient had a history of recurrent abortion (P0+12) with no laboratory evidence of antiphospholipid syndrome. The heavy bleeding was associated with palpitation, headache, and dizziness. She had a history of recurrent pulmonary embolism. The abdominal examination was unremarkable. The initial laboratory test showed hemoglobin of 6.5 g/dl and mean corpuscular hemoglobin of 25.5 picograms.

**Figure 1:**
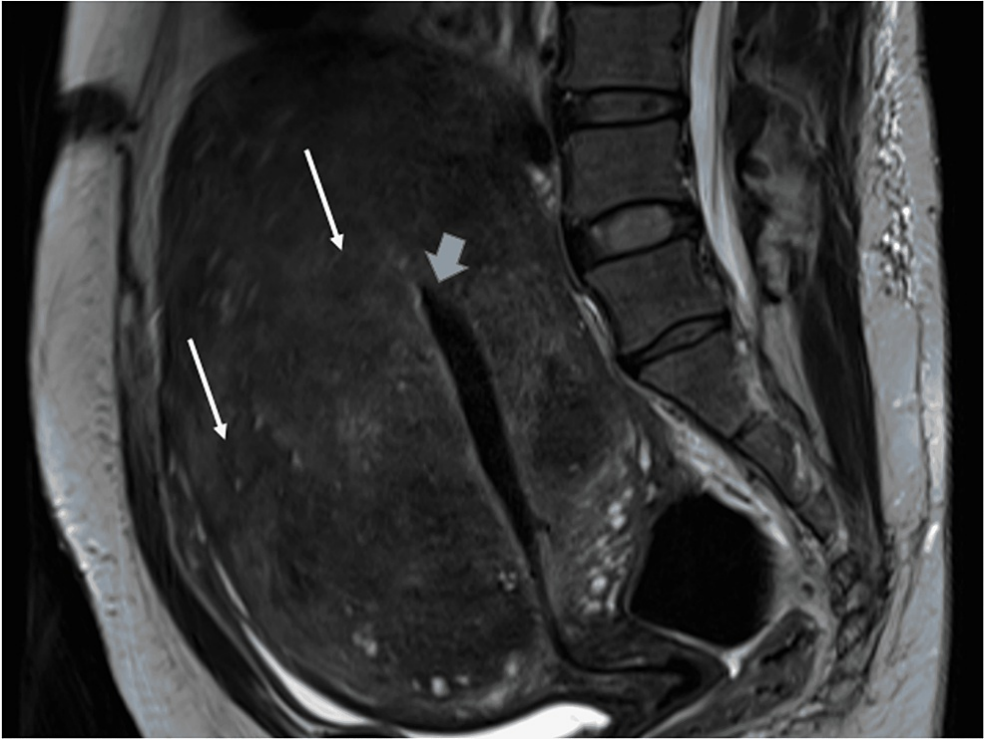
Pelvic MRI Sagittal section of MRI pelvis showing enlarged uterus with multiple small cysts within the myometrium (white arrows). The endometrial cavity is highlighted by the blue arrow, which shows thickened endometrium.

Pelvic magnetic resonance imaging (Figure 1) revealed an enlarged anteverted uterus with a diffusely thickened junctional zone with multiple small cystic changes and hemorrhage, which are associated with features of adenomyosis. The endometrial thickness measured 0.8 cm. Both adnexa were unremarkable.

Based on the clinical and radiological evaluation, subtotal hysterectomy with left salpingo-oophorectomy was performed, and the specimen was sent for histopathological examination. On gross examination, the intact specimen was composed of the uterus, left fallopian tube, and ovary. The cut section of the uterus showed a thin endometrial lining with 0.2 cm thickness. The myometrium was thickened and measured 12.5 cm × 12 cm × 5 cm. Its cut surface showed a whirly tan surface with small cystic and slit-like spaces. The fallopian tube measured 5 cm in its maximum dimension. The left ovary was atrophic.

**Figure 2:**
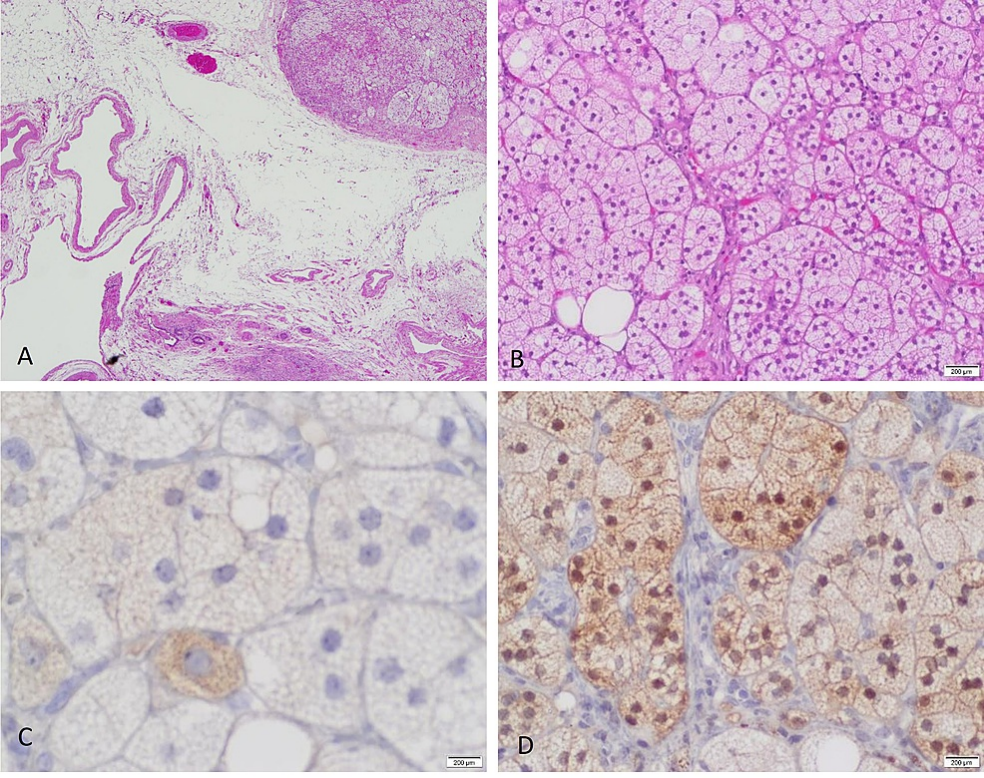
Adrenal cortical rest of the fallopian tube (H&E and immunostaining) This figure shows the histopathologic examination by hematoxylin and eosin (H&E) stains and immunohistochemistry studies. (A) Examination revealed a well-circumscribed nodule of adrenal cortical tissue located in the hilar region of the fallopian tube. (B) This panel shows the cytological features of this nodule with no infiltration into the adjacent tissue and no necrosis. The nucleus is central with fine chromatin and inconspicuous nucleoli. The cytoplasm is abundant and clear (H&E, 4x and 20x). (C) Inhibin immunostaining showing granular cytoplasmic positivity (40x). (D) Calretinin immunostaining showing nuclear and cytoplasmic reactivity (20x).

On microscopic evaluation, the hematoxylin and eosin (H&E) stain of the myometrium showed features of adenomyosis with benign atrophic overlying endometrium. Incidentally, during the examination of the fallopian tube, a well-circumscribed nodule was identified and composed of a collection of polygonal cells with distinct cellular borders. The nucleus was central and showed a normal nuclear to cytoplasmic ratio and fine granular chromatin. The cytoplasm was clear to amphophilic. All of these features were typical of the adrenal cortex’s normal appearance (Figure 2). No atypical features, such as necrosis, atypical mitosis, cytological atypia, or infiltrative growth, were noted. Immunohistochemical markers were used, including cytokeratin, inhibin, calretinin, and synaptophysin, to rule out metastatic renal cell carcinoma and heterotopia of ovarian hilus cells. The cells showed reactivity for inhibin and calretinin (Figure 2), confirming that the described nodule was an ectopic adrenal cortical rest.

## Discussion

EAT is considered an incidental histological finding with a 1% incidence among adult populations [5]. Embryologically, the adrenal cortex develops from coelomic mesoderm during the fourth to sixth week of gestation [6]. It is most commonly reported in males, specifically in the testis. While it is very rare in females, when it does appear, it shows a predilection for the broad ligament [2]. Other relevant and reported sites include the kidneys, peri-adrenal tissue, retroperitoneal fat, ovaries, uterus, and testis. These rests are exclusively composed of cortical cells without adrenal medullary cells [7].

Microscopically, EAT is easy to diagnose based on classic histological appearance. Immunohistochemical studies need to differentiate between the adrenal rests from metastatic clear cell renal cell carcinoma, displaced ovarian luteinized theca cells, and heterotopia of ovarian hilus cells. The adrenal cortex is typically immunoreactive for Melan-A (MART1), inhibin, synaptophysin, and calretinin and has a high nuclear positivity (86%) to antisteroidogenic factor-1 (anti-SF-1). While metastatic clear renal cell carcinoma is negative for these markers and positive for CD10, AE1/AE3, and carbonic anhydrase, the ovarian hilus cell heterotopia of the fallopian tube shows similar immunopositivity to adrenal cortical cells with the difference of the additional positivity for chromogranin, which is negative in adrenal cortical cells [8]. In our case, we noted immunoreactivity to inhibin and calretinin that was compatible with the published data.

We reviewed the English literature in PubMed, Google Scholar, and Ovid using the following terms: “ectopic adrenal tissue,” “ectopic adrenal rest,” and “fallopian tube.” We found only two cases reported in fallopian tubes. The first case was reported by Tingi and Ogah in 2018 in a 48-year-old female with heavy abnormal bleeding. The patient underwent a total abdominal hysterectomy with bilateral salpingo-oophorectomy. After a microscopic examination of the left fallopian tube, they found the adrenal cortical tissue, which they designated as adrenal ectopic tissue [7]. As in our case, the adrenal ectopic tissue identified in the left fallopian tube of the patient with heavy bleeding was explained by the presence of massive adenomyosis.

The second case was reported by Tzigkalidis et al. in 2021 in a 37-year-old female diagnosed with ectopic pregnancy in the right fallopian tube. They noticed a minute nodule, firm, yellowish in color, and measuring 2 mm, which they labeled microscopically as adrenal ectopic tissue. They found this nodule to be positive by immunohistochemistry for MART1, synaptophysin, calretinin, and inhibin-A. As in our case, the EAT showed immunoreactivity for the calretinin and inhibin-A [8].

To date, the presence of EAT has no known major clinical significance and no related symptoms or functional properties have been previously documented. However, it is crucial to recognize EAT because it may develop the same pathologies that arise from the adrenal gland in its normal location, such as hyperplasia, adrenal insufficiency, and neoplastic transformation.

## Conclusions

EAT is an uncommon finding, and discovery of the ectopic tissue in the fallopian tube is extremely rare. EAT has no clear clinical significance. Nevertheless, precise evaluation should be performed to rule out other differential diagnoses and primary pathological abnormalities in ectopic tissue.

## References

[1] Anderson JR, Ross AH (1980). Ectopic adrenal tissue in adults. Postgrad Med J.

[2] Ors F, Lev-Toaff A, O'Kane P, Qazi N, Bergin D (2007). Paraovarian adrenal rest with MRI features characteristic of an adrenal adenoma. Br J Radiol.

[3] Souverijns G, Peene P, Keuleers H, Vanbockrijck M (2000). Ectopic localisation of adrenal cortex. Eur Radiol.

[4] Alimoradi M, El-Helou E, Sabra H, Azaki R, Khairallah M, Matta N (2020). Ectopic adrenal gland in an adult inguinal hernial sac: a case report. Int J Surg Case Rep.

[5] Senescende L, Bitolog PL, Auberger E, Le Bian AZ, Cesaretti M (2016). Adrenal ectopy of adult groin region: a systematic review of an unexpected anatomopathologic diagnosis. Hernia.

[6] Aidan C (2012). Histology for Pathologists. https://www.amazon.com/Histology-Pathologists-Published-Lippincott-Hardcover/dp/B00HQ1EO3U.

[7] Tingi E, Ogah J (2018). Ectopic adrenal rest cells of the fallopian tube: a case report and review of the literature. J Obstet Gynaecol.

[8] Tzigkalidis T, Skandalou E, Manthou ME, Kolovogiannis N, Meditskou S (2021). Adrenal cortical rests in the fallopian tube: report of a case and review of the literature. Medicines (Basel).

